# C-C Chemokine Receptor 7 Promotes T-Cell Acute Lymphoblastic Leukemia Invasion of the Central Nervous System via β2-Integrins

**DOI:** 10.3390/ijms25179649

**Published:** 2024-09-06

**Authors:** Cesar I. Cardona, Alondra Rodriguez, Vivian C. Torres, Anahi Sanchez, Angel Torres, Aaron E. Vazquez, Amy E. Wagler, Michael A. Brissette, Colin A. Bill, Charlotte M. Vines

**Affiliations:** 1 Department of Biological Sciences, The University of Texas at El Paso, El Paso, TX 79936, USAcabill@utep.edu (C.A.B.); 2 Public Health Sciences, The University of Texas at El Paso, El Paso, TX 79968, USA; awagler2@utep.edu

**Keywords:** C-C Chemokine Receptor 7, antagonist, integrin

## Abstract

C-C Chemokine Receptor 7 (CCR7) mediates T-cell acute lymphoblastic leukemia (T-ALL) invasion of the central nervous system (CNS) mediated by chemotactic migration to C-C chemokine ligand 19 (CCL19). To determine if a CCL19 antagonist, CCL19_8-83_, could inhibit CCR7-induced chemotaxis and signaling via CCL19 but not CCL21, we used transwell migration and Ca^2+^ mobilization signaling assays. We found that in response to CCL19, human T-ALL cells employ β2 integrins to invade human brain microvascular endothelial cell monolayers. In vivo, using an inducible mouse model of T-ALL, we found that we were able to increase the survival of the mice treated with CCL19_8-83_ when compared to non-treated controls. Overall, our results describe a targetable cell surface receptor, CCR7, which can be inhibited to prevent β2-integrin-mediated T-ALL invasion of the CNS and potentially provides a platform for the pharmacological inhibition of T-ALL cell entry into the CNS.

## 1. Introduction

The American Cancer Society estimates that there will be 62,770 new cases of leukemia and 23,670 leukemia-related deaths in the United States this year [1]. Acute lymphoblastic leukemia (ALL) accounts for 75% of cancer cases among children, with a peak incidence between 2 and 5 years of age [1]. T-cell acute lymphoblastic leukemia (T-ALL) is an aggressive form of cancer that develops in approximately 25–30% of ALL cases [1,2,3]. It has been reported that between 50 and ~73% of human pediatric T-cell acute lymphoblastic leukemias (T-ALLs) express activating NOTCH1 transcription factor mutations [4,5,6,7]. These activating mutations of Notch1 have been observed in ~73% of T-ALL relapse patients as well. The presence of these mutations implicates a role for activated Notch1 in the etiology and or progression of human T-ALL.

Using a mouse model, it was shown that T-ALL oncogene Notch1-activating mutations are upstream in a pathway leading to elevated expression of the G protein-coupled receptor C-C Chemokine Receptor 7 (CCR7) and that enhanced CCR7 levels promoted T-ALL invasion of the central nervous system (CNS) [8]. Furthermore, transplantation of the CCR7 expressing human pediatric T-ALL cell line, CCRF-CEM, into immunocompromised Rag2^−/−^ Il-2Rγ^−/−^ mice increased the leukemia load and CNS involvement when compared to a human pediatric T-ALL cell line, DND41, that lacked CCR7 expression, in which no CNS infiltration was observed. In these mice, however, ectopic expression of CCR7 was able to increase the leukemic burden and CNS penetration, suggesting that the activation of CCR7 alone was sufficient for CNS invasion [8].

CCR7 induces chemotactic migration in response to two ligands, C-C chemokine ligand 19 (CCL19) and CCL21 [9,10]. CCL19 is upregulated during T-ALL invasion of the CNS, where it is thought to promote entry of the leukemic cells past the blood–brain barrier (BBB) [8]. The BBB is formed by endothelial cells that use zonule occludins (ZO-1) and claudins to form tight junctions that facilitate the assembly of cells into intact monolayers [11] while playing crucial roles in maintaining the integrity of the BBB [12,13]. The BBB prevents the exit of substances from circulation into the CNS. Once T-ALL has breached the BBB and entered the CNS, mediastinal irradiation does not provide clear therapeutic benefits, leading physicians to use intrathecal chemotherapy prophylactically to attempt to prevent the survival of T-ALL that enters the CNS [2]. Current treatment protocols for pediatric T-ALL incorporate the use of glucocorticoids, vincristine, asparaginase, methotrexate, 6-mercaptopurine, and at times anthracyclines, cytosine arabinoside, and cyclophosphamide [14]. The use of these intrathecal drugs often has long-term toxic effects, and therefore, a better understanding of the molecular mechanisms that promote the invasion of T-ALL into the CNS could provide platforms for the development of targeted pharmaceuticals to prevent CNS entry and spare children from toxic therapies.

CCR7 can be directly inhibited using a well-defined murine CCL19 antagonist, CCL19_8-83_ [15]. This peptide antagonist of CCR7 is an N-terminal truncation of CCL19 that is naturally expressed in the body. Under homeostatic conditions, CCR7 is sulfonated on tyrosine residues that promote interaction with CCL19 [16,17,18]. X-ray crystallographic structure analysis of the human antagonist of CCL19, CCL19_7-70_, revealed that in contrast to CCL21, the recognition sites for CCL19 are within positively charged and hydrophobic amino acids within the C-terminus of CCL19 and within the 40’s loop [16,17]. These sulfonated tyrosines are thought to be important for the binding of both CCL19 and CCL21 and may be conserved in many chemokines and make useful targets for potentially novel drugs [18,19,20].

In this study, we used CCL19_8-83_ as a CCL19 antagonist to block the migration of T-ALL into the CNS. We used the well-described PR/SET Domain 14 (PRDM14) mouse on an Mx-1 Cre background (PRDM14;Mx-1-Cre), allowing for the induction of T-ALL, which expresses high levels of Notch1 [21,22]. We determined that the T-ALL cells in PRDM14;Mx-1-Cre mice express elevated levels of CCR7. Since T-cells can migrate via β1 or β2 integrins on fibronectin-coated polycarbonate membranes [23], we examined the contribution of CCR7 to migration via both integrins. Additionally, using an in vitro model of intact microvascular endothelial cells, we found that CCR7 promotes the migration of T-ALL cells across microvascular endothelial cell membrane monolayers via the activation of β2 integrins. Furthermore, we showed that this migration can be blocked by antagonizing CCR7. Using CCL19_8-83_ prophylactically in vitro at 5-day intervals over the course of twenty days following the induction of leukemia in the PRDM14;Mx-1-Cre mouse, in which the T-ALL cells express high levels of Notch1 mutations [21,22], we found that treatment CCL19_8-83_ leads to an increase in the lifespan of mice that have been induced to develop T-ALL. These studies provide an important platform for a potential therapeutic that may be used instead of commonly used, toxic intrathecal chemotherapies as a prophylactic to block the migration of T-ALL into the CNS during the 2.5–3-year treatment protocol for T-ALL.

## 2. Results

### 2.1. CCL19_8-83_ Inhibits CCL19-Induced CCR7 Internalization and Signaling but Not CCL21

**Figure 1:**
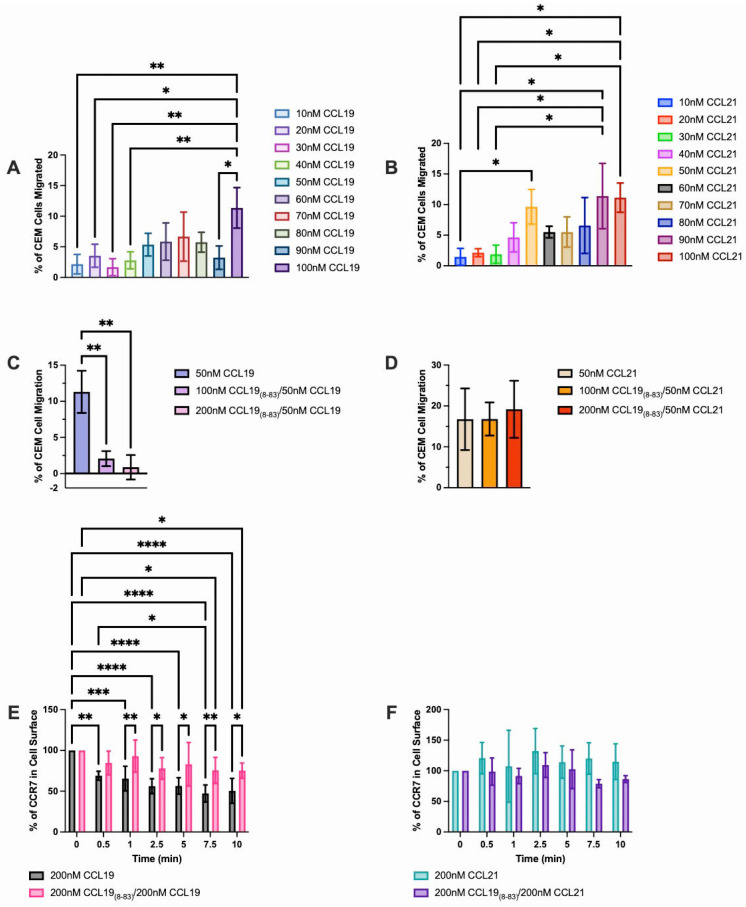
Characterization of CCL19, CCL21, and CCL19_8-83_ in CCR7-CEM cells. Chemotaxis of CCRF-CEM cells was assessed using a Boyden Chamber assay. (**A**) CCL19 or (**B**) CCL21 at the indicated concentrations or 50 nM (**C**) CCL19 or (**D**) CCL21 was added to the lower wells. CCRF-CEM cells (**A**,**B**) alone or (**C**,**D**) with CCL19_8-83_ at the indicated concentration in the upper wells. Cells were allowed to migrate for 3 h across fibronectin-coated, 5 µm pore polycarbonate membranes. The percentage of cells migrated [(number of cells loaded/number of cells in bottom well) × 100] is shown. CCRF-CEM cells were induced to internalize (**E**) CCR7/CCL19 or (**F**) CCR7/CCL21 for the indicated times ± CCL19_8-83_ and CCR7 remaining on the cell surface was assayed by flow cytometry. Data points represent the mean ±SD for *n* = 3+ independent experiments. * *p* < 0.05, ** *p* < 0.01, *** *p* < 0.001, **** *p* < 0.0001 as determined using one-way ANOVA (**A**–**D**) or two-way ANOVA (**E**,**F**).

To define the concentration of CCL19_8-83_ needed to block migration to CCL19, we initiated our studies using CCRF-CEM, human pediatric T-ALL cells. For these studies, cell migration was measured across a fibronectin-coated polycarbonate transwell membrane at chemokine concentrations ranging from 10 nM to 100 nM (Figure 1). CCL19-induced chemotaxis of CCRF-CEM cells was observed at the lowest concentration of 10 nM and the number of cells responding increased until peak migration was observed at 70 nM. We observed a similar dose response to CCL21, where maximal migration was observed at 100 nM. We observed a similar dose response to CCL21, where maximal migration was observed at 100nM (Figure 1B). Moreover, we determined that transwell migration of CCRF-CEM cells to CCL19 (Figure 1C), but not CCL21 (Figure 1D) was inhibited in the presence of 100nM or 200nM CCL19_8-83_.

To determine if CCL19_8-83_ blocked CCL19-mediated internalization of CCR7 without affecting CCL21, we used human CCRF-CEM T-ALL cells in a flow cytometry-based internalization assay (Figure 1). When stimulated with CCL19 alone, CCRF-CEM cells internalized 31% of surface CCR7 at 0.5 min and 53% of available CCR7 over 7.5 min. In support of our previous observations, CCL21 failed to internalize, and, indeed, intracellular stores appeared rapidly on the surface within 30 s as the levels slightly increased. To determine if CCL19_8-83_ specifically antagonized only CCL19 or both ligands, CCRF-CEM cells were pre-exposed to 200 nM CCL19_8-83_, followed by incubation in the presence of 200 nM CCL19 or CCL21. We found that in the presence of equimolar concentrations of ligand and antagonist, CCL19_8-83_ blocked the internalization of CCL19 but not of CCL21 (Figure 1). Moreover, transwell migration of CCRF-CEM to CCL19 but not CCL21 was inhibited in the presence of CCL19_8-83_ (Figure 1).

**Figure 2:**
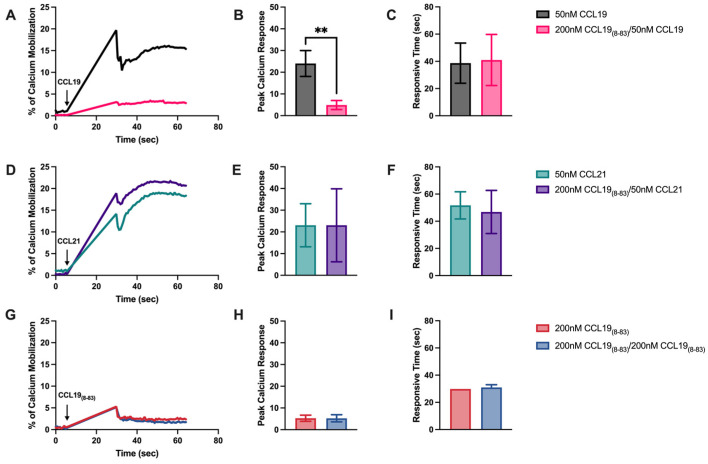
CCL19_8-83_ blocks CCL19-induced calcium mobilization. (**A**,**D**,**G**) CCRF-CEM cells were pre-loaded with Fluo-4 AM at 37 °C, rinsed, and pre-incubated with CCL19_8-83_ for five minutes before being stimulated with CCL19, CCL21, or CCL19_8-83_. Calcium mobilization was normalized to the ionomycin control maximum; data are plotted as the percentage of ionomycin control. (**B**,**E**,**H**), the maximal peak normalized percentage of Ca^2+^ mobilized and (**C**,**F**,**I**), time (seconds) to reach peak Ca^2+^ mobilization. Data points represent the mean ± SD of *n* = 3 independent experiments. ** *p* < 0.01 (unpaired two-tailed Student’s *t*-test).

To determine if CCL19_8-83_ disrupted not only receptor internalization but also signaling in CCRF-CEM T-ALL via CCR7, we used Ca^2+^ mobilization assays. Cells were pre-loaded with the fluorescent calcium indicator Fluo-4AM, pre-incubated in the presence of 200 nM CCL19_8-83_, and induced to mobilize Ca^2+^ in the presence of CCL19 or CCL21 (Figure 2). The data were normalized to the maximum Ca^2+^ released in the presence of ionomycin. CCL19_8-83_ significantly blocked the peak Ca^2+^ response in the presence of CCL19 (Figure 2A,B) without affecting the mobilization of Ca^2+^ in the presence of CCL21 (Figure 2D,E). CCL19_8-83_ alone did not mobilize Ca^2+^ (Figure 2G,H).

### 2.2. CCL1_8-83_ Prevents Migration of T-ALL across a Human Endothelial Cell Monolayer

**Figure 3:**
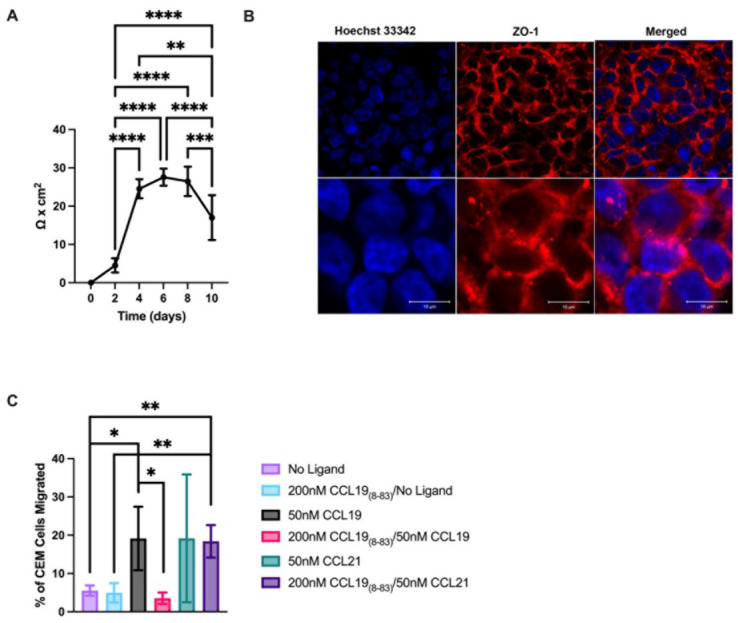
CCL19_8-83_ inhibits CCL19-induced chemotaxis of CCRF-CEM across an intact HBEC-5i monolayer. (**A**) Trans-Endothelial Electrical Resistance (TEER) was measured every two days over 10 days. Data points represent the mean ± SD; *n* = 9. (**B**) HBEC-5i were plated on glass coverslips and allowed to form monolayers over several days. Cells were permeabilized and stained with anti-ZO-1-Alexa Fluor 647 (direct conjugate) and nuclei were stained with Hoechst and imaged at 40X (upper panel) or digitally zoomed (lower panel) to observe tight junctions. (**C**). T-ALL cell trans-endothelial cell migration to CCL19 but not CCL21 was inhibited in the presence of CCL19_8-83._ Cells were pre-incubated in the presence of CCL19_8-83_ and allowed to migrate to CCL19 across an HBEC-5i monolayer for 16 h, and cells that migrated to the lower well were counted. Data is the mean ± SD of *n* = 3 independent experiments. * *p* < 0.05, ** *p* < 0.01, *** *p* < 0.001, **** *p* < 0.0001. Scale bar = 10 µm.

To investigate the CCR7-induced molecular mechanisms that promote T-ALL CNS invasion, we used a human brain microvascular endothelial cell line, HBEC5i, to model the BBB. To establish an intact monolayer, we used 24-well transwell inserts and allowed the cells to grow for 4–8 days (see Methods Section 4). We measured an average maximal TEER of 27.6 Ω × cm^2^ (Figure 3). To assess the integrity of the monolayer and the localization of zonule occludins to the edges of the cells where they form tight junctions, we stained for ZO-1 (Figure 3) and found that the ZO-1 localized to the junctions between cells. By day 10, the integrity of the monolayer began to deteriorate as reflected in a decrease in the TEER (Figure 3); therefore, all the transmigration studies were carried out between days 4 and 8.

To determine if CCL19_8-83_ could be used to inhibit the migration of CCRF-CEM into the CNS, we used the BBB model (Figure 3). Intact HBEC-5i monolayers were established, CCL19, CCL21, or media (control) was added to the lower wells at the optimal concentration for migration (See Figure 1), and cells were allowed to migrate over 16 h. CCRF-CEM cells were layered on the apical surface of the HBEC-5i monolayer. We found that 19.6% of cells migrated to CCL19, but when cells were pretreated with CCL19_8-83_, only 3.6% of the cells were able to migrate (Figure 3). In contrast, in the presence of CCL21, cell migration remained unchanged independent of the presence of CCL19_8-83._ In the absence of CCR7 ligands, the CCRF-CEM cells did not penetrate the HBEC-5i monolayer (Figure 3C). We concluded that CCL19_8-83_ at 200 nM should be able to inhibit CCL19-medicated CNS invasion of T-ALL across the BBB but not CCL21.

**Figure 4:**
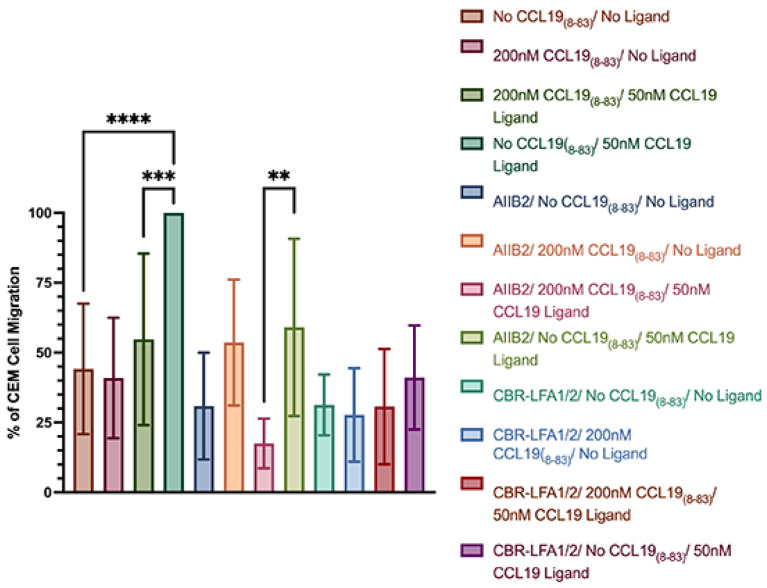
CCL19_8-83_ inhibits β2 integrin-mediated T-ALL migration across a cerebral microvascular endothelial cell monolayer. CCRF-CEM cells were pre-incubated with 10 µg/mL AIIB2 (anti-β1 integrin) ± CCL19_8-83_ or 10 µg/mL CBR-LFA1/2 (anti-β2 integrin) for five minutes prior to loading in the top well. Cells were allowed to invade and transmigrate over 16 h, and migrated cells normalized to the number of cells that migrated to CCL19 alone. *n* = 3 independent experiments. *n* = 3 independent experiments. ** *p* < 0.01, *** *p* < 0.001, **** *p* < 0.0001. (unpaired two-tailed Student’s *t*-test).

To determine if CCL19_8=83_ inhibited the CCR7-induced activation of integrins during T-ALL transmigration of the BBB, we inhibited integrin function during the transwell migration assays using integrin-specific antibodies (Figure 4). In the presence of anti-β1 integrin function-blocking antibodies, cells failed to migrate independent of the presence of CCL19 or CCL19_8-83_, indicating that T-cells can spontaneously migrate via β1 integrins independent of CCL19. In contrast, in the presence of β2 integrin antibodies, T-ALL cells failed to migrate through polycarbonate membranes in the absence of a ligand, while in the presence of CCL19, T-ALL cell migration was blocked in the presence of CCL19_8-83_. T-ALL cells migrated in the presence of CCL19, which indicates that β1 integrins can promote migration across a polycarbonate membrane in response to stimulation with CCL19.

CCRF-CEM migration to CCL19 across a microvascular endothelial monolayer can be blocked by CCL19_8-83_ (Figure 4). Since trans-endothelial migration employs β1 and β2 integrins, it was unclear which integrin was inhibited by CCL19_8-83._ To determine if CCL19_8-83_ blocked β1 integrin-mediated migration, we examined CCRF-CEM cell migration in the presence of anti-β1 integrin antibodies. We found the CCRF-CEM migration to CCL19 was not significantly different ± anti-β2 integrin antibodies independent of the presence of CCL19_8-83_. In contrast, CCL19 promoted β1-integrin-mediated invasion/transmigration of endothelial cells in the presence of CCL19 and anti-β1 integrin antibodies but not when CCL19_8-83_ was present. From these results, we infer that CCL19 promoted β2-integrin-mediated migration, which was inhibited in the presence of anti-β1 integrin antibodies and significantly reduced migration to below-baseline levels.

#### CCL19_8-83_ Prophylactic Treatment Substantially Extends Survival in a Mouse Model of T-ALL

**Figure 5:**
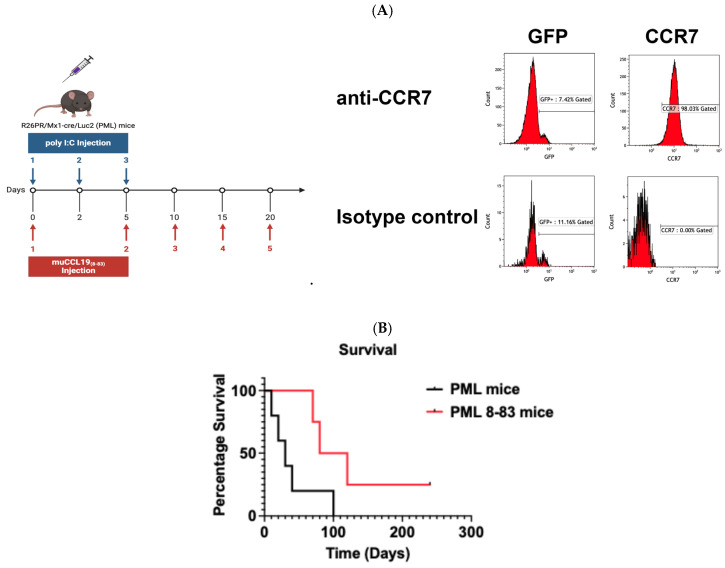
Invasion of the CNS by GFP-expressing T-ALL blasts is inhibited by CCL19_8-83_ injections and substantially improves survival. (**A**) T-ALL was induced by injecting 200 µg of polyinosinic:polycytidylic acid (poly I:C) to stimulate the production of interferons and the activation of Cre in interferon-responsive cells. PBMCs were isolated by submandibular bleed, and RBCs were lysed and the PBMCS stained for CCR7. GFP+ cells were analyzed for CCR7 expression. (**B**) Following induction of leukemia on day 0 with polyI:C, mice were injected with CCR7 antagonist 200 CCL19_8-83_ on days 0, 5, 10, 15, and 20. *p* = 0.058.

To examine the effect of CCL19_8-83_ on survival, we used the well-characterized PRDM14-inducible model. In this mouse, the PRDM14 locus has been cloned downstream from the *lox*P-STOP-*lox*P cassette, within a *ROSA26* promoter. To determine if these cells express CCR7, we stained the surface and analyzed the cells by flow cytometry (Figure 5). We found that of the cells that expressed GFP, 57 ± 15.4% expressed CCR7 as well. Although the cells were either CD4+ or CD8+, CD8+ cells outnumbered CD4+ at a ratio of 2.8:1. Mice treated with CCL19_8-83_ survived substantially longer than mice that were not treated. Specifically, untreated mice survived an average of 40 ± 15.8 days compared to an average of 127.5 ± 39.0 days for mice treated with CCL19_8-83_. These results suggest that CCL19_8-83_ inhibition of signaling in mice with T-ALL that express CCR7 substantially enhances survival.

## 3. Discussion

Approximately 15% of childhood and 25% of adult ALL are T-ALL [24], which has a higher risk of induction failure, CNS infiltration, and relapse compared to B-cell ALL. T-ALL is a disease of the improper development of T-cells and in most cases is linked to the activation of Notch1 mutations and the overexpression of CCR7 [8]. The expression of CCR7 in T-ALL is sufficient to cause pediatric T-ALL invasion of the CNS in response to chemotaxis to CCL19. Our study examined the CCL19 agonist, CCL19_8=83_, a derivative of a non-toxic peptide, CCL19, which is normally expressed in the body [25]. In this study, we found that CCL19_8-83_ inhibits CCL19 at a 75-fold lower concentration than was previously published in in vitro assays [15], at a level that is tolerable to mice. At this concentration, we found a significant increase in the survival rate of mice induced to develop leukemia from an average of 40 days post-induction to 127 days (Figure 5).

During cell migration, chemokine receptors bind to and internalize chemokine ligands to promote directed migration [26,27]. Differences in the internalization of CCR7 ligands have been reported in naïve and activated CD4+ and CD8+ T-cells, dendritic cells, and CCR7 transfected cells [28]. Indeed, we found that in human HuT78 T-ALL cells, CCL19 rapidly induced arrestin-3 mediated internalization of approximately 80% of surface CCR7, while CCL21 internalization was arrestin-3 independent and internalized less than 20% of the available CCR7 [29]. In this study, we found that CCL19_8-83_ specifically inhibited the internalization of CCR7 via CCL19 while having no effect on signaling through CCL21 (Figure 1, Figure 2, Figure 3 and Figure 4). Indeed, we observed slight increases in the levels of CCR7 expressed on the surface of cells following treatment with CCL21, which were likely due to release from intracellular stores [30].

Although CCR7 is required, it is unclear how or where T-ALL cells can enter the CNS. The BBB, consisting of endothelial cells, pericytes, capillary basement membranes, and astrocyte foot processes held together by tight junctions and enzyme barriers, is a major protective layer determining what substances or cells can leave the blood and enter the CNS [31]. To establish the minimum level of CCL19_8-83_ required to inhibit CCL19-induced signaling, we used in vitro transmembrane migration assays (Figure 1). We were able to determine that at 100 nM CCL19_8-83_, the antagonist can selectively inhibit migration to CCL19 but not CCL21. Since CCL21 is an important ligand for promoting the migration of naïve T-cells to lymph nodes, maintaining CCL21-mediated lymph node chemotaxis of immune cells will allow for the maintenance of homeostatic trafficking and function within lymph nodes.

Downstream of CCR7 binding to a ligand, we have shown that CCL19 and CCL21 activate phospholipase Cγ1 (PLCγ1) [32,33], which can mediate cleavage of phosphatidylinositol (4,5)-bisphosphate (PIP_2_), leading to the production of IP_3_ and the resultant mobilization of Ca^2+^ from intracellular stores in lymphocytes [28]. Using Ca^2+^ mobilization assays [34], we found that CCL19_8-83_ selectively blocked CCL19- but not CCL21-induced Ca^2+^ mobilization. These results further supported the targeted and selective inhibition of CCL19 but not CCL21 signaling by CCL19_8-83_.

Transmembrane migration assays provided a basis for using the doses that we used in subsequent studies both in vitro and in vivo. Our studies used both fibronectin-coated transwells and a simplified barrier model consisting of microvascular endothelial cells that formed tight junctions. The TEER measurements made of our HBEC5i monolayers, averaging 27.6 Ω × cm^2^, were characteristic of an intact monolayer, as were the presence of zonule occludins within the cell junctions (Figure 3). In our studies, CCRF-CEM migrated across the barrier formed by these cells in response to CCL19 and CCL21. Only transmigration to CCL19 could be blocked by CCL19_8-83_; this antagonist had no effect on CCL21-induced transmigration.

Characterization assays (Figure 1 and Figure 2) provided a starting point for the doses that we used in subsequent studies both in vitro and in vivo. Our studies used both fibronectin-coated transwells and a simplified barrier model consisting of cerebral microvascular endothelial cells that formed tight junctions. The TEER measurements made of our HBEC-5i monolayers, averaging 27.6 Ω × cm^2^ were characteristic of an intact monolayer, as were the presence of zonule occludins within the cell junctions (Figure 3). In our studies, CCRF-CEM cells migrated across the barrier formed by these cells in response to CCL19 and CCL21. Only trans-migration to CCL19 could be blocked by CCL19_8-83_; this selective antagonist did not affect CCL21-induced transmigration.

Of potential clinical relevance, previous studies by our lab showed that β2-integrin can mediate the migration of T-cells into the CNS, and in these studies, we disrupted migration using nanoparticles that were bound to a polymer [35,36]. In the former and current studies, disrupting the migration of T-cells into the CNS by disrupting β2-integrin binding directly with nanoparticles or indirectly through antagonizing a G protein-coupled receptor (GPCR), CCR7, limited disease. It comes as no surprise that more than 475 drugs that are currently on the market target GPCRs [37] given their targetability. We have identified a novel role for T-ALL therapy that may provide a platform for the development of pharmaceuticals in the future.

Using the PRDM14/MX-1 Cre mouse allowed us to examine the effects of CCL19_8-83_ on an immune-replete mouse. When crossed with MX-1 Cre mice, injection of the synthetic double-stranded RNA polyI:C leads to the expression of Cre recombinase in cells that have receptors for type I interferons [38]. Following Cre recombination, the expression of PRDM14 and GFP is activated by the constitutive *ROSA26* promoter. The induced T-ALL cells were reported to express a mutated Notch1 and invade the CNS [21,22]; however, the CCR7 status was unknown. We found that these T-ALL cells express CCR7 (Figure 5) and that the survival of these mice can be substantially increased by prophylactic treatment with CCL19_8-83_ (Figure 5). In addition, we found that CD8+ cells outnumbered CD4+ at an average ratio of 2.8:1. However, the differences in the ratio of CD4+:CD8+ did not affect mouse survival.

Since the T-ALL cells expressed CCR7, it is tempting to speculate that the enhanced lifespan of treated mice is due to CCL19_8-83_ blocking the CCR7-mediated migration of T-ALL cells into the mouse CNS, which is currently under investigation in our lab. We have shown that when stimulated, CCR7 can also promote the exit of immune microglia from the CNS [39]. In our current study, independent of exposure to CCL19_8-83,_ T-ALL in mice express CCR7. However, since the mice survived on average more than 100 days after the final administration of the CCL19_8-83_, it is unclear to what extent exposure of the T-ALL to a CCR7 function-blocking peptide that disrupts β2 integrin adhesion may affect the survival of the T-ALL cells within the animal. In human patients, the survival of T-ALL requires a combination of α_L_β_2_ integrin and α_4_β1 integrin-mediated adhesion to bone marrow stroma [40]. A loss of β2 integrin adhesion correlated with a loss of leukemia cell survival in the bone marrow. While we observed no effect of CCL19_8-83_ on β1-integrin-mediated adhesion, we did find that the antagonist blocked β2 integrin-mediated transmigration. In addition, there is some evidence that like B-cell ALL, T-ALL may migrate from the bone marrow directly into the CNS.

In the future, it will be important to determine the effects of CCL19_8-83_ on T-ALL cell survival in vivo. Regardless of the mechanism of CCL19_8-83_ actions, it is evident that the use of an inhibitor of CCR7, which belongs to the large G protein-coupled receptor family that makes up about a third of all FDA-approved drugs, can be targeted with significant in vivo survival benefits. A significant complication of pediatric T-ALL is CNS invasion where the leukemic cells can hide during standard chemotherapy regimens, resulting in disease recurrence. Given the improved survival of the CCL19_8-83_ treated T-ALL mice, clinically, CCL198-83 has potential as a prophylactic treatment for pediatric T-ALL patients, keeping T-ALL cells and possibly other diseased T-cells in the periphery where they can be killed by effective chemotherapies.

## 4. Materials and Methods

Cell Culture CCRF-CEM T-ALL cells were a generous gift from Dr. Iannis Aifantis (New York University, Langone Health) and were maintained in complete Roswell Park Memorial Institute 1640 media (RPMI 1640) supplemented with 10% heat-inactivated fetal bovine serum (∆FBS) (cRPMI) and 2 mM L-glutamine. HBEC5i [41] (ATCC, CRL-3245; Manassas, VA, USA) were purchased from ATCC and maintained in Dulbecco’s Modified Eagle Medium:Nutrient Mixture F-12 (DMEM/F-12) supplemented with 1 X Endothelial Cell Growth Supplement (Sciencell, Carlsbad, CA, USA), 10% ∆FBS, and 2 mM L-glutamine (cDMEM/F-12) on tissue culture plastic that was pre-coated with a 0.1% gelatin solution (Sigma-Aldrich, Burlington, MA, USA). The cells were maintained at 37 °C in a 5% CO_2_ humidified atmosphere.

Reagents CCL19 and CCL21 were purchased from R&D labs, and muCCL19_8-83_ was purchased from Pepmic, Suzhou, China. RPMI 1640 was purchased from Clontech (San Jose, CA, USA), heat-inactivated fetal bovine serum was purchased from R&D Systems (Minneapolis, MN, USA), and ionomycin from Millipore Sigma (Burlington, MA, USA).

Transwell Chemotaxis Assays Chemotactic migration of CCRF-CEM was measured using Neuro Probe AP48 transwell chambers, as described by us [32]. Briefly, the lower wells of the NeuroProbe AP48 were loaded with 30 µL of CCL19 or CCL21 at the indicated concentrations diluted in cRPMI-1640 or serum-free RPMI (*sf*RPMI). The lower wells were covered with a 5 µm pore polycarbonate filter that had been coated with 10 µg/mL human plasma fibronectin (Sigma-Aldrich (Burlington, MA, USA), FC010-10MG). Two hundred thousand CCR7-CEM cells were pre-incubated with either *sf*RPMI or CCL19_(8-83)_ and added to the upper chamber. The cells were induced to migrate for 3 h at 37 °C in a 5% CO_2_ humidified incubator. The migration of the cells was assessed by determining the total number of cells in the lower chambers using duplicate wells for each point. The migration of the cells was assessed by counting the total number of cells in the lower well. Each experiment was performed in duplicate, and the results were normalized to the number of cells that migrated with the ligand alone. All the values were normalized to the number of cells that migrated to the media alone (transmigration) or CCL19 (integrin-blocking) and calculated as [(cells migrated/cells loaded) × 100].

Flow Cytometry Assays. The assays were conducted as described by us [29], with the following modifications. Briefly, CCRF-CEM T-ALL were resuspended at 2 × 10^7^ cells per ml in RPMI1640 and pre-incubated ± muCCL19_8-83_ for 5 min, ligand-adjusted to 200 nM of CCL19 or CCL21, and allowed to internalize CCR7 for the indicated times. Following receptor internalization, the cells were plunged into 10-fold ice-cold cRPMI/2% bovine serum albumin and allowed to rest on ice for 10 min. The cells were centrifuged (90× *g* for 5 min) and resuspended in anti-hCCR7-PE-Cy7 (BD Pharmingen Cat#557648; San Diego, CA, USA) or isotype control (IgG2a-PE-Cy7 BD Pharmingen Cat#557855) and incubated at 4 °C for 30 min. The cells were washed 3 × in PBS/2% BSA. The cells were analyzed for forward scatter, and cell surface levels of CCR7 were analyzed by flow cytometry (Beckman Coulter Gallios (Irving, TX, USA) and analyzed using Kaluza software (Version 2.2.1). CCR7-PE-Cy7 binding, which was not inhibited by either CCL19 or CCL21, on the surface of unstimulated cells was defined as 100% at t = 0. Statistical significance was determined using the paired *t* test. The data were analyzed using GraphPad Prizm10 software (Version 10.1.2).

Calcium Mobilization assays CCRF-CEM cells were resuspended at 1.5 × 10^7^ cells/mL in Hanks Balanced Salt Solution (HBSS)/Fluo-4 AM (Invitrogen, F14201) for 45 min with gentle inversion every 5 min. CCL19_8-83_ was added for five minutes to the blocked cells, and the cells were stimulated with either CCL19 or CCL21. The levels of mobilized Ca^2+^ were normalized to the levels mobilized in the presence of ionomycin, which was considered 100%. Fluorescence levels were measured on a Fluoroskan Ascent FL (Thermo Scientific, Waltham, MA, USA).

Trans-Endothelial Electrical Resistance (TEER) of Cerebral Microvascular Endothelial Cell Monolayers. HBEC-5i cells were seeded at 6.6 × 10^4^ cells/mm^2^ in cDMEM:F-12 on 24-well transwell inserts (Corning 3472) that had been pre-coated with 10 µg/mL fibronectin (Sigma-Aldrich, FC010-10MG). The integrity of the cerebral microvascular endothelial monolayer was determined by measuring TEER every two days using Millicell-ERS (Millipore, MEERS00002( Billerica, MA, USA) over 10 days. The TEER values were corrected by subtracting the TEER of a blank transwell insert without cells from the TEER measurements. The reported TEER values were normalized to the membrane surface area as Ω × cm^2^. The average TEER values observed, 27.6 Ω × cm^2^, are characteristic of an intact monolayer [41,42].

Immunofluorescent Analysis of Tight Junctions HBEC-5i cells were plated on poly-L-Lysine (EMD Millipore, A-005-C)-coated coverslips in cDMEM:F-12 at 4 × 10^5^ cells/mL, allowed to grow to 100% confluency, and fixed with 4% formaldehyde in PBS for 20 min at RT. The coverslips were blocked/permeabilized with blocking buffer (2% bovine serum albumin/PBS/0.1%Triton X-100) for 1 h and stained with anti-ZO-1-Alexa Fluor^®^ 647 conjugate (Cell Signaling Technologies #98225 (Danvers, MA, USA) for 2 h at a 1:100 dilution in blocking buffer. The nuclei were stained with Hoechst Dye at 1:100 in PBS for 5 min at RT. The coverslips were mounted onto glass slides in ProLong Gold Antifade (Invitrogen P36934 (Waltham, MA, USA), cured overnight, and imaged (Zeiss LSM 700 Confocal, Dublin, CA, USA).

Transmigration of CCRF-CEM through Cerebral Microvascular Endothelial Cell Monolayers. HBEC-5i cells were seeded in cDMEM-F-12 media at 6.6 × 10^4^ cells/cm^2^ on 24-well transwell inserts (Corning 3472) and grown for 4–8 days [41] to form an intact cerebral microvascular endothelial cell monolayer as determined by TEER. CCR7-CEM cells were pre-incubated in cRPMI ± 200 nM CCL19_8-83_, and 7.5 × 10^5^ CCRF-CEM cells were added to the top of each intact cerebral microvascular endothelial cell monolayer. To determine the integrins involved in transmigration across a microvascular cell monolayer, CCRF-CEM cells were pre-incubated with 10 µg/mL AIIB2 (anti-β1 integrin) ± CCL19_8-83_ or 10 µg/mL CBR-LFA1/2 for five minutes prior to loading into the top well. The cells were allowed to invade and transmigrate over 16 h through the monolayer at 37 °C/5%CO_2_ in a humidified atmosphere ± CCR7 ligands. At 16 h, the cells that had migrated to the lower compartment were counted. For the transmigration assays, the values given were normalized to the number of cells loaded, and for the integrin studies, the values were normalized to the number of cells that migrated to CCL19 alone, in the absence of function-blocking antibodies.

Mice ROSA26-*lox*P-STOP-*lox*P-Prdm14-IRES-eGFP (*R26PR*) mice were a generous gift from Dr. Monica Justice at the Hospital for Sick Children, Toronto [21,22]. B6.Cg-Tg(Mx-1-Cre)1Cgn/J and FVB.129S6(B6)-Gt(ROSA)26SOR^tm(Luc)KaeI^/J mice were purchased from The Jackson Labs. The mice were housed in the University Laboratory Animal Care Facility at The University of Texas at El Paso. All the mouse manipulations were carried out under the approval of the Institutional Animal Care and Use Committee (IACUC Protocol #A201310 approved 2/2022).

Induction of Leukemia R26PR mice were crossed with B6.Cg-Tg(Mx-1-Cre)1Cgn/J mice to produce R26PR/Mx-1 Cre mice. These mice were bred to FVB.129S6(B6)-Gt(ROSA)26SOR^tm(Luc)KaeI^/J to produce R26PR/Mx-1 Cre/Luc2 mice. The animals genotyped were confirmed using tissues collected during the placement of the identification chips. To induce leukemia, mice were treated as described in [21,22]; eight-week-old mice were injected with 200 µg polyI:polyC, a TLR3 agonist [43], every other day for a total of 3 injections [21,22]. The CCL19_8-83_-treated mice were injected with 200 µg/mouse of CCL19_8-83_ ip on days 10, 15, and 20. The mice were weighed every 3–4 days starting at day 21 until they reached 6 months of age or lost greater than 20% of body weight, or their body condition score reached level 2, at which point the mice were euthanized. The numbers of mice used were based on the in vivo published study, which describes the role of CCR7 in T-ALL migration across the BBB [8].

## Data Availability

All the data in this manuscript can be accessed through CMV or CAB upon request.
